# "Vasopressor Support Sparing Strategies": a Concept to be Incorporated as a Paradigm in the Treatment of Vasodilatory Shock

**DOI:** 10.21470/1678-9741-2019-0600

**Published:** 2019

**Authors:** Paulo Roberto B. Evora, Domingo M. Braile

**Affiliations:** 1 Editor-in-Chief Interim - BJCVS Faculdade de Medicina de Ribeirão Preto da Universidade de São Paulo (FMRP-USP), Ribeirão Preto, SP, Brazil.; 2 Editor-in-Chief - BJCVS Faculdade de Medicina de São José do Rio Preto (FAMERP), São José do Rio Preto, SP, Brazil and Universidade de Campinas (UNICAMP), Campinas, SP, Brazil.

## The BJCVS Highlight

Unacceptably high rates of mortality in critically ill patients, including those under cardiopulmonary bypass (CPB), are a reality that keeps itself through time, despite advances in pharmacology and technology. Shock treatment initiated with fluid resuscitation strategies and administration of adrenergic vasopressor agents in nonresponsive patients to restore arterial pressure and protect the microcirculation. High catecholamine (norepinephrine) dosing requirements are necessary to achieve targeted hemodynamic goals, increasing the risk of vasopressor-induced adverse events. In addition, catecholamines are associated with well-known side effects, including increased myocardial oxygen consumption and development of arrhythmias, which are two compromising conditions of good evolution even of elective cardiac surgeries^[1]^.

Catecholamines are predominantly used in supraphysiological doses to overcome the consequences of pathological inflammatory shock. However, these adrenergic agents cause direct organ damage and have multiple harmful biological effects on immune, metabolic and coagulation pathways, with negatively patient outcomes. Andreis & Singer^[2]^ appropriately called this situation "the schizophrenic 'Jekyll-and-Hyde' catecholamines characteristics in critical illness", as they are both necessary for survival although detrimental in excess. It is clear that this Jekyll-and-Hyde drama was based on the microcirculatory detrimental of high and prolonged use of catecholamines. Therefore, it is imperative to find ways of protecting microcirculation against the deleterious effects of catecholamines, justifying the motivation of this Editorial about "Vasopressor Support Sparing Strategies" as a concept to be incorporated as a paradigm in the treatment of vasodilatory shock^[2]^.

Few randomized studies exist to guide clinical management and hemodynamic stabilization in patients who do not respond to the standard approach (fluid resuscitation and norepinephrine). Many adjuvant therapies, such as hydrocortisone (high doses used in the 1970's), thiamine and ascorbic acid, have been suggested to increase blood pressure in severe shock and should be considered when combined vasopressor therapy is needed^[3]^. Nowadays, vasopressin and methylene blue seems to be the most commonly used drugs as on option for association with high doses of norepinephrine (NE). Vasopressin acts through membrane receptors and methylene, by blocking the NO/cGMP pathway, "crosstalking with the amine-dependent PGI2/cAMP pathway"^[4]^.

Based on almost 25 years of experimental and clinical experience our opinion is that methylene blue (MB), at present, may be the best, safest, and cheapest option. Even in the absence of definitive multicentric studies, experimental studies suggest a potential protective role of MB in microcirculation. In an experimentally-induced septic shock model in rats, only the combination of NE + MB restored mean arterial pressure to control levels by the end of the three-hour experiment^[5,6]^.

Finally, the current concept should be illustrated by the above mentioned "Jekyll-and-Hyde" analogy. Some remarks should be mentioned based on the "Vasopressor Support Sparing Strategies" paradigm:

1. What is the best drug considering the pharmacological mechanism (membrane receptors, endothelium-dependent mechanisms...)?
2. Precocious "window of opportunity" or even always adopt the support sparing strategies?
3. Search for novel vasopressor agents, such as synthetic human angiotensin II, which would increase blood pressure and reduce the need for high doses of catecholamine vasopressors.
4. Optimistically, if possible, seek new vasopressors that increase the arterial blood pressure without microcirculatory damage.

There are few effective rescue therapies for established refractory shock, which emphasizes the importance of aggressive intervention before refractory shock develops, including earlier initiation of rational combination vasopressor therapy.

## Articles in this Issue

This issue of BJCVS presents a blind peer-reviewed selection of 20 papers that were selected by order of acceptance: 11 original papers, 2 review articles, 1 special article, 2 multimedia and 4 selected case reports. In response to our editorial efforts, two Letters to the Editor have been included in this issue.

**Paulo Roberto B. Evora** https://orcid.org/0000-0001-9631-946X ^1^Editor-in-Chief Interim - BJCVS Faculdade de Medicina de
Ribeirão Preto da Universidade de São Paulo (FMRP-USP),
Ribeirão Preto, SP, Brazil.**Domingo M. Braile** https://orcid.org/0000-0001-7704-2258 ^2^Editor-in-Chief - BJCVS Faculdade de Medicina de São
José do Rio Preto (FAMERP), São José do Rio Preto, SP, Brazil
and Universidade de Campinas (UNICAMP), Campinas, SP, Brazil.
